# From signal transduction to protein toxins—a narrative review about milestones on the research route of *C. difficile* toxins

**DOI:** 10.1007/s00210-022-02300-9

**Published:** 2022-10-07

**Authors:** Klaus Aktories

**Affiliations:** Institute of Experimental and Clinical Pharmacology and Toxicology, Medical Faculty, University of Freiburg, Albertstr. 25, 79104 Freiburg, Germany

**Keywords:** G proteins, Bacterial protein toxins, *C. difficile* toxin TcdB, *C. difficile* ADP-ribosyltransferase CDT, Pseudomembranous colitis, Toxin receptors, Toxin up-take

## Abstract

Selected findings about *Clostridioides difficile* (formerly *Clostridium difficile*) toxins are presented in a narrative review. Starting with a personal view on research about G proteins, adenylyl cyclase, and ADP-ribosylating toxins in the laboratory of Günter Schultz in Heidelberg, milestones of *C. difficile* toxin research are presented with the focus on toxin B (TcdB), covering toxin structure, receptor binding, toxin up-take and refolding, the intracellular actions of TcdB, and the treatment of *C. difficile* infection.

A lecture by Klaus Aktories on May 6, 2022, delivered on the occasion of the mini-symposium in honor of Prof. Dr. med Günter Schultz (1936–2021).

## Starting point: molecular pharmacology in Heidelberg

This narrative review about toxin research has its starting point in Heidelberg, when I joint the laboratory of Günter Schultz at the Institute of Pharmacology of the University of Heidelberg in 1978. This had been an extremely exciting time in science and especially in pharmacology. “Signal transduction” had been the great theme in pharmacology, aiming to explain the control, regulation and impact of cellular signal pathways involved in transduction of the activities of hormones, neurotransmitters and drugs, following their binding to cell membrane receptors. This general research intention has resulted in enormous advances of knowledge about drug actions on the molecular level and shaped a research, which has been called *biochemical pharmacology* and, later, *molecular pharmacology*. In Günter Schultz’ laboratory regulation of adenylyl cyclase and guanylyl cyclase were the main topics, mainly represented by the work of the group leaders Karl-Heinz Jakobs and Eyke Böhme and supported by the group of Franz Hofmann (Gerzer et al. 1981; Schultz et al. 1982; Aktories et al. 2019). Without question, this work in the Schultz laboratory has turned out to have outstanding impact on the development of biochemical and molecular pharmacology in Germany.

## Cholera toxins and pertussis toxin to study G proteins

In late seventies, G-proteins have already been recognized as coupling factors between receptors and adenylyl cyclase, which were controlled by GDP release, GTP binding and GTP hydrolysis (Pfeuffer 1977). Cholera toxin (CT) was shown to persistently activate the Gs proteins by ADP-ribosylation and its potential role as a tool in signal transduction research was obvious (Cassel and Pfeuffer 1978). In the early eighties, pertussis toxin (PT) (Ui 1984) was introduced and it turned out that this toxin, which blocked the activity of Gi proteins by ADP-ribosylation was extremely instrumental for studies on the inhibitory Gi proteins, which was the main topic of Karl-Heinz Jakobs in the Schultz laboratory (Kather et al. 1983; Aktories et al. 2019). Using these toxins as excellent tools to manipulate G-protein-dependent signaling, we extended our studies by introducing *Clostridium botulinum* C2 toxin, which was shown to have major effects on cell morphology. I used the same assays for C2 toxin as for CT and PT and found a large modification of a protein in the molecular mass range of G proteins (e.g., ~ 40 kDa). However, it turned out to be the cytoskeleton protein actin, a fact, which was eventually verified by my own group at the Rudolf-Buchheim-Institute (director at that time Ernst Habermann) of the University of Gießen (Aktories et al. 1986).

## *Clostridium botulinum* C3 toxin, a tool to study small GTPases of the Rho family

Purification of C2 toxin from a certain strain of *Clostridium botulinum* revealed another protein with ADP-ribosylating activity (Aktories et al. 1987). This led to the discovery of C3 toxin, which turned out to ADP-ribosylate a new family of small GTPases called Rho proteins (Rösener et al. 1987; Chardin et al. 1989; Vogelsgesang et al. 2007). It was immediately clear that C3 toxin is an excellent tool to unravel the action of Rho proteins. With this toxin in my bag, I went into the laboratory of Alan Hall (at that time at the Chester Beatty Institute in London) for a sabbatical (Aktories et al. 1989; Aktories and Hall 1989; Paterson et al. 1990). This was the starting point of a great discovery story performed by the laboratory of Alan Hall (and numerous others,^1^ e.g., Shu Narumiya’s group in Kyoto), eventually resulting in the elucidation of the roles and functions of Rho proteins (Jaffe and Hall 2005; Narumiya et al. 2009). While initially the research on the roles of Rho proteins focused on their actions as master regulators of the cytoskeleton, with time it became clear that many essential cellular functions are regulated by Rho proteins. Moreover, the discovery of Rho proteins was also a milestone in toxin research, because it turned out that numerous toxins and bacterial effectors act on Rho proteins (and related small GTPases). It was found that Rho proteins are not only ADP-ribosylated (Aktories et al. 2004; Lang et al. 2010; Visvikis et al. 2010; Aktories 2011), but also glucosylated (Just et al. 1995a, 2001; Busch and Aktories 2000; Jank et al. 2013), deamidated (Flatau et al. 1997; Schmidt et al. 1997), proteolytically cleaved (Shao et al. 2003), and AMPylated (Yarbrough et al. 2009). In addition, an incredibly large number of bacterial effectors hijack the switch function of Rho proteins by acting as mimics of Rho regulatory proteins, including their GAP, GEF and GDI functions (Cherfils and Zeghouf 2013; Hicks and Galan 2013).

This lecture will focus on large clostridial glucosylating toxin with the prototypes *Clostridioides difficile* (formerly known as *Clostridium difficile*) toxins A and B. These toxins are produced by *C. difficile*, a spore-forming anaerobe Gram + bacterium, which is the cause of a spectrum of diseases, ranging from self-limiting diarrhea (antibiotics-associated diarrhea) to pseudomembranous enterocolitis with severe complications like bowel perforation, toxic megacolon and death (Kelly and LaMont 1998, 2008; Bartlett 2006; Viswanathan et al. 2010; Abt et al. 2016). Major concern in disease management is the recurrent colitis (with increasing fatality rates), which occurs in ~ 25% of colitis patients after initial successful treatment. In 2017, estimated 224,000 cases of hospitalized patients and 12,800 deaths were reported in the USA (https://www.cdc.gov/drugresistance/biggest-threats.html#cdiff) (Slimings and Riley 2014; Lessa et al. 2015; Abt et al. 2016). In most cases, *C. difficile* infection is the consequence of treatment with antibiotics. Almost all antibiotics are able to cause *C. difficile* infections, probably most important are clindamycin, cephalosporins, carbapenems, amoxicillin plus clavulanic acid and fluoroquinolones (Slimings and Riley 2014).

## What is the initiating pathomechanism of the infection?

**Fig. 1:**
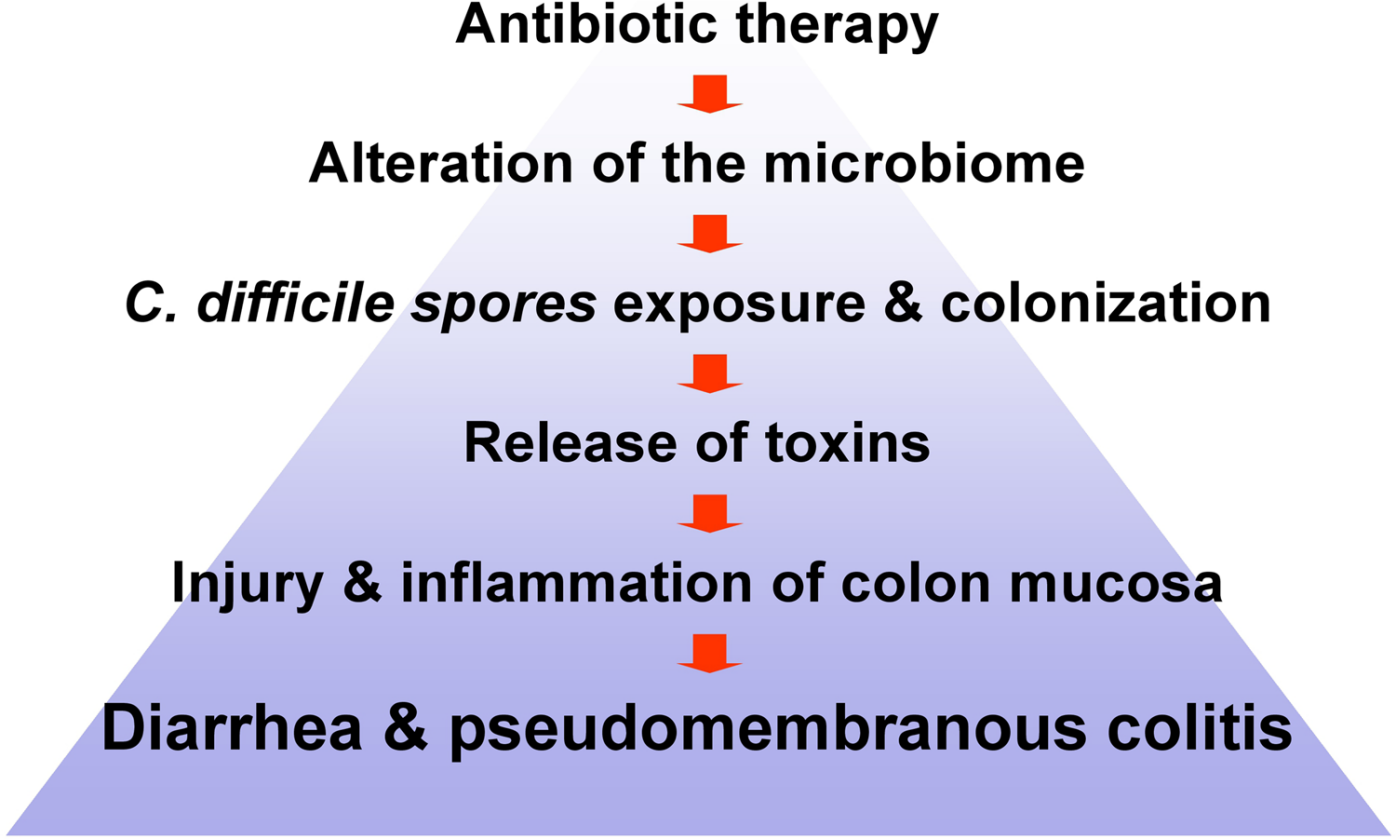
Antibiotics-induced *C. difficile* infection (CDI). The various steps involved in CDI are exhibited

**Fig. 2:**
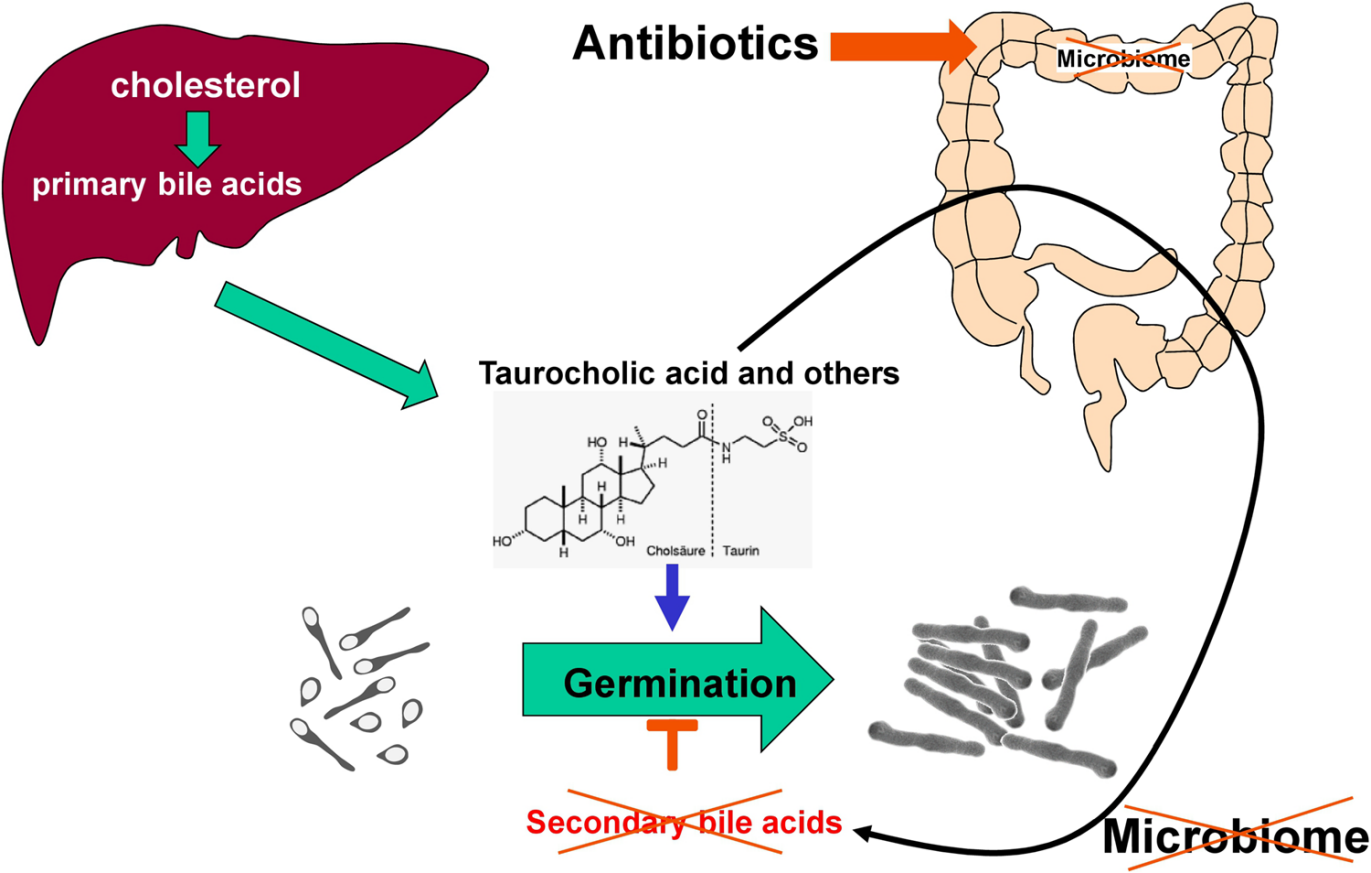
Effects of bile acids on germination of *C. difficile* spores. Germination of *C. difficile* spores is enhanced by primary bile acids and bile acid conjugates (like taurocholic acid). Secondary bile acids, which are produced from primary bile acids by the gut microbiome, inhibit germination. Therefore, antibiotics, which damage the gut microbiome, enhance germination of spores

Treatment with antibiotics severely alters the microbiome of the gut (Fig. 1). When *C. difficile* spores are in the surrounding, oral uptake followed by germination and proliferation follows. Eventually, the bacteria produce toxins, which damage the epithelium of the colon and induce inflammation and necrosis. Destruction of the physiological microbiome plays a crucial role in these events. Under normal conditions, *C. difficile* spores are not able to germinate efficiently in the human gut. Germination of spores are controlled by several factors including bile acids and short fatty acids (Lawler et al. 2020; Shen 2020; Yuille et al. 2020) (Fig. 2). Primary bile acids like cholic acid and chenodeoxycholate are conjugated with taurine (and glycine) in the liver and secreted into the gut where especially taurocholic acid and taurochenodeoxycholate act as strong enhancers of germination. Secondary bile acids are formed from primary bile acids and bile acid conjugates by microbiome-derived enzymes, like bile salt hydrolases (BSH) and 7α-dehydroxylase. These enzymes induce hydrolysis of bile acid conjugates and formation of deoxycholic acid (DCA) and lithocholic acid (LCA), which inhibits germination and growth of *C. difficile*. Thus, alteration of the gut microbiome has major consequences on germination of *C. difficile* spores.

Infection with *C. difficile* causes typical histopathological changes, which are characterized by damage of gut mucosa initially with volcano- or mushroom-like localized necrotic areas, which are formed by disposition of fibrin with granulocytes and eventually resulting in formation of large necrotic areas with typical pseudomembranes (Counihan and Roberts 1993). This damage of colon tissue exclusively depends on protein toxins produced by *C. difficile*. Three toxins are produced by *C. difficile*: two “major” so-called large clostridial glucosylating toxins (LGTs), toxin A (TcdA), and toxin B (TcdB) and a third ADP-ribosylating toxin called *C. difficile* transferase (CDT). The pathophysiological role of CDT is still not finally clarified (Voth and Ballard 2005; Genth et al. 2008; Aktories et al. 2017a; Orrell and Melnyk 2021; Kordus et al. 2022).

## Structural feature of LGTs

**Fig. 3:**
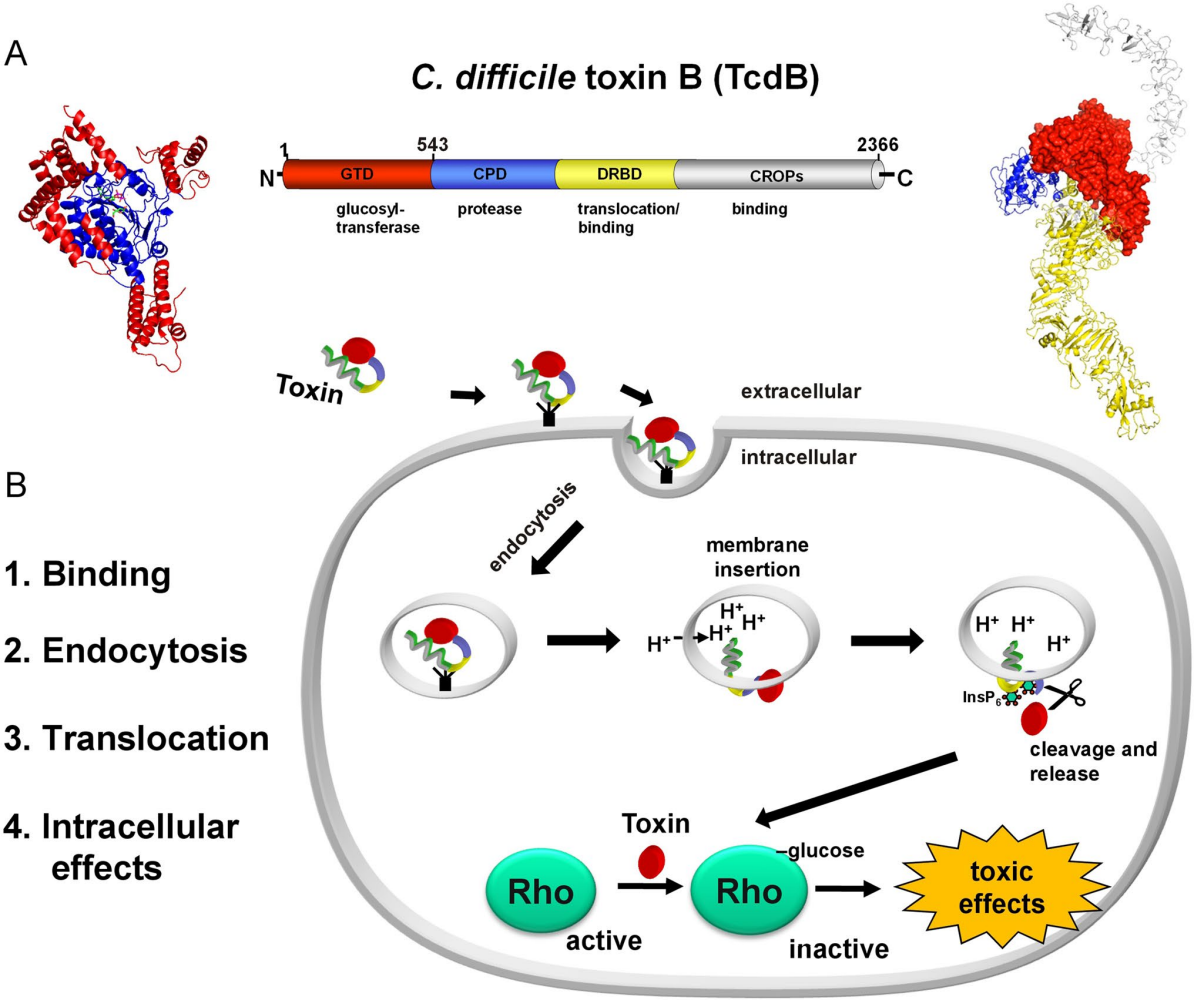
*C. difficile* toxin B structure and action. A. Left part: the structure of the glucosyltransferase domain of *C. difficile* toxin B (TcdB) is shown (In dark blue, is the catalytic core of the glucosyltransferase depicted and in red peripheral helices are given. Middle part: Scheme of the 4 functional domains of TcdB. At the N-terminus is the glucosyltransferase domain (GTD, red), it follows the inherent cysteine protease (blue), the delivery and binding domain (DRBD, yellow) and at the C-terminus the CROPs domain (gray), which is also involved in binding. Right part: Crystal structure of the complete TcdB. Domains are colored as in the linear scheme depicted in the middle part. B. left part: The typical steps of the actions of an intracellularly acting toxin are listed. Right part: The toxin (e.g., TcdB) binds to its receptor and is endocytosed, at low pH of endosomes the toxin is able to insert into the endosomal membrane. In the cytosol, the cysteine protease domain is activated by InsP6 (inositol hexakisphosphate), thereby the glucosyltransferase domain (GTD) is released. GTD modifies Rho proteins by mono-*O*-glucosylation and blocks the regulatory actions of these switch proteins. Pictures were designed using (10.2210/pdb7v1n/pdb) and (10.2210/pdb2BVL/pdb) by PyMol

TcdA and TcdB both share four domains (Jank and Aktories 2008; Pruitt et al. 2010; Aktories et al. 2017a; Kordus et al. 2022). At the N-terminus, the glucosyltransferase domain (GTD) is located, which represents the biologically active part of the toxin (Fig. 3). It follows a cysteine protease domain (CPD) responsible for the autoproteolytic processing of the toxins. The delivery and receptor binding domain (DRBD) is involved in translocation (delivery) and binding of the toxin. The C-terminus represents the combined repetitive polypeptide (CROP) domain, also involved in cell surface binding. The crystal structure of the isolated GTD from TcdB was first reported in 2005 (Reinert et al. 2005) (Fig. 3). The structures of the isolated CROPs domain (Ho et al. 2005) and of the protease domain (Pruitt et al. 2009) were solved in 2005 and 2009, respectively. The 3D structure of full-length TcdB has not been solved until 2019 (Chen et al. 2019) likely due to the high flexibility of the CROPs domain (Fig. 3).

The four-domain structure is shared by most LGTs, which are produced by various species related to *C. difficile,* and motivated us, some years ago, to introduce the ABCD model of the toxins (A = active glucosyltransferase; B (CROPs) = Binding, C = Cutting (CPD), D = Delivery (DRBD) (Jank and Aktories 2008). However, the LGT from the *Clostridium perfringens* toxin TpeL possesses only 3 domains missing the CROPs domain (Aktories et al. 2017a).

## Receptor binding of *C. difficile* toxins

**Fig. 4:**
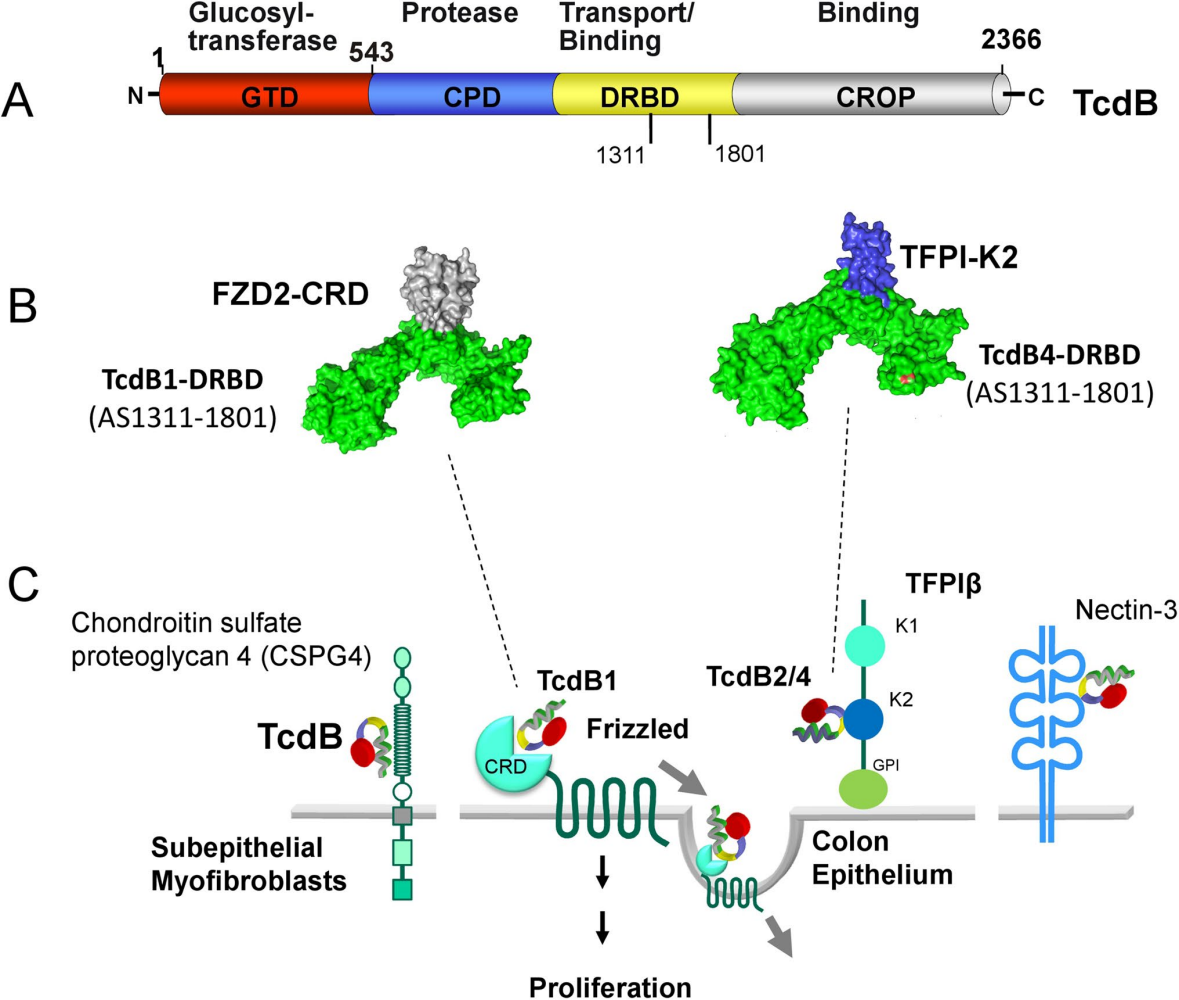
Receptors and binding of TcdB. A. Schema of the 4 domain structure of TcdB. the binding region for receptor interaction with the receptors frizzled (FZD) and tissue factor pathway inhibitor (TFPI) is indicated (residues 1311–1801). B. Surface view of the crystal structure of part of the delivery/receptor-binding domain (DRBD) of TcdB in complex with the receptors FZD and TFPI. The toxin subtype TcdB1 binds to the cysteine rich domain of frizzled (FZD1-CRD), while the toxin subtype TcdB4 binds to the Kunitz domain 2 of TFPI (TFPI-K2). The binding areal of both toxin subtypes is almost identical. C. Various receptors of TcdB are shown. Chrondroitin sulfate glycoprotein 4 (CSPG4) binds both toxin subtypes but is not located on the intestinal epithelium. The heptahelical frizzled receptor binds TcdB1 with its cysteine rich domain (CRD). Frizzled is a receptor for Wnt ligand and involved in proliferation. TFPI binds with its Kunitz 2 domain TcdB4. Nectin-3 has been also identified as TcdB receptor. Its pathophysiological role is not clear. Picture was designed on the bases of (10.2210/pdb6C0B/pdb) and (10.2210/pdb7V1N/pdb) by PyMol

At least 4 steps are essential for the action of LGTs: 1. Receptor binding, 2. endocytosis, 3. translocation/delivery, and 4. the intracellular activity (Fig. 3). Studies on receptor binding has taken surprising turns. Up to recently, two major cell membrane receptors were described for TcdB: Chrondroitin sulfate proteoglycan 4 (CSPG4) (Yuan et al. 2015) and heptahelical receptors of the Frizzled family (FZD 1, 2 and 7) (Tao et al. 2016) (Fig. 4). In addition, nectin-3 (LaFrance et al. 2015) and low-density lipoprotein receptor-related protein 1 (LRP1) (Guo et al. 2022) have been reported as potential receptors, but their function in toxin action is not clear. CSPG4 is not present at the surface of epithelial gut cells but rather on subepithelial myofibroblasts, this receptor is probably not crucial for initial interaction of TcdB with the gut epithelium. Thus, FZD seemed to be the most prominent and pivotal receptor for TcdB. This view is supported by the fact that Frizzled is involved in Wnt signaling, which plays a pivotal role in the regenerative potency of crypt stem cells of the gut (Polakis 2007). Repair and rapid regeneration of gut epithelium cells depend largely on crypt stem cells, the damage of which by *C. difficile* toxins could explain the action of TcdB on gut epithelium (Chen et al. 2018). In addition, it has been reported that at least in organoids TcdB fragments (without glucosylating activity) block Wnt signaling pathways by interacting with the FZD receptor (Tao et al. 2016). The interaction of TcdB with FZD has been studied in greater detail. TcdB binds to FZD1, 2, and 7. The N-terminal cysteine-rich domain of FZD, which is involved in binding of ligands of the Wnt family, is also responsible for the binding of TcdB. Interestingly, it was observed that TcdB exploits a free fatty acid (palmitoleic acid) as the co-receptor to engage FZDs (Chen et al. 2018). A similar fatty acid, which is a lipid modification of Wnt, is also crucial for the binding of Wnt ligands to FZD.

## High genetic variability of *C. difficile*

The concept of FZD as a receptor for TcdB was challenged by the finding that some toxin variants do not interact with FZD (Mileto et al. 2020). In this respect, it is important to mention that during the last 15 years it became clear that *C. difficile* is characterized by high genetic variability (Hunt and Ballard 2013). The special impact of certain *C. difficile* strains for severity of infections got broader attention with reports on the outbreak of *C. difficile* infection by strain NAP1/027/BI (North American pulse field type 1 (NAP1), ribotype 027, restriction-endonuclease type BI) and their association with increased morbidity and mortality (McDonald et al. 2005). Further intensive studies on the diversity *C. difficile* strains revealed that at least 5 major *C. difficile* clades have to be considered, which largely differ in virulence (Stabler et al. 2006). Initially, the so-called hypervirulent strains were characterized by fluoroquinolone resistance, high toxin production and synthesis of CDT (Loo et al. 2005). Later, a large number of “hypervirulent” strains were recognized and importantly, different toxin types were characterized, which paralleled the genetic variability. Recently, 8–12 major TcdB subfamilies (subtypes) were identified with more than 200 different toxin members, which are all at least ~ 85% identical in amino acid sequence (Mansfield et al. 2020; Shen et al. 2020). The abovementioned hypervirulent strain NAP1/027/BI belongs to clade 2 of *C. difficile*, whereas the prototype toxin from strain VPI belongs to clade 1.

## New receptors for *C. difficile* TcdB

Studies performed with different types of TcdB showed that these subtypes target stem cells but apparently not via FZD. What is then the toxin receptor? Recently, genome-wide CRISPR/Cas9-dependent screenings revealed that tissue factor pathway inhibitor (TFPI) is a colonic receptor for TcdB subtypes from *C. difficile* clade 2 (Fig. 4). Particularly, TcdB4, which is another clade 2 toxin, depends on TFPI (Luo et al. 2022).

TFPI is an anticoagulant protein produced primarily by endothelia and megakaryocytes (Broze and Girard 2012). It inhibits coagulation factor Xa, the function of the TF-FVIIa complex and the initial prothrombinase complex. Two alternatively spliced isoforms, TFPIa and TFPIb, are known. TFPIa is a secreted protein, which is bound on cell membranes and in plasma. It consists of three protease-inhibiting Kunitz domains (K1–3). TFPIb has two Kunitz domains (K1 and K2) domains. Additionally, it has a GPI anchor for membrane insertion. Kunitz 1 domain of TFPI binds coagulation factor VIIa, and the K2 domain (TFPIK2) binds and inhibits factor Xa (Broze and Girard 2012). TcdB4 binds both TFPI isoforms at their K2 domain and thereby blocks interaction with FXa (Luo et al. 2022). Structural analysis revealed that the Kunitz 2 domain of TFPI binds to exactly the same region of TcdB4 (receptor-binding interface covers residues 1,431–1,606 of TcdB4), which is involved in TcdB1 for binding to FZD (Chen et al. 2018) (Fig. 4). Moreover, phylogenetic analysis of various TcdBs revealed 2 major toxin classes, with class I RBIs common in TcdB1, TcdB3, and TcdB5, which bind FZD and class II interfaces for binding of TcdB2, TcdB4, TcdB6, and TcdB7, which mainly represent clade 2 *C. difficile* toxins. Interestingly, the same region of the related lethal toxin (TcsL) from *Paeniclostridium sordellii* (formerly *Clostridium sordellii*), sharing ~ 76% identity with TcdB1, is involved in binding to Semaphorin A and B, the receptors of TcsL (Lee et al. 2020; Tian et al. 2020). The data suggest that LGTs get their variability by intragenomic recombination and reveal an evolutionary mechanism for switching receptors.

## Translocation of TcdB

The translocation of LGTs is still not well understood. In 2001, we reported that TcdB is able to form pores at low pH in target cells (Barth et al. 2001). Two years later, we showed that not the whole toxin but only the N-terminal glucosyltransferase is delivered into the cytosol (Pfeifer et al. 2003). Obviously, the toxin is processed by proteolytic cleavage. It was a major step forward, when, in 2007, it was recognized that inositol hexakisphosphate (InsP6) was essential for processing of the toxin (Reineke et al. 2007). At the same time, we identified the protease domain of LGTs and its dependence on InsP6 (Egerer et al. 2007, 2009). This finding resulted in the four-domain model (ABCD model) of LGTs (Jank and Aktories 2008). It is generally accepted that after endocytosis, the toxin-receptor complex reaches the low pH compartment of endosomes, from where the glucosyltransferase domain together with the protease domain is translocated into the cytosol (Fig. 3). Here, the CPD is activated by InsP6, resulting in release of the GTD. However, the mechanism of the delivery of the glucosyltransferase into the cytosol is still not clarified.

## Refolding of TcdB by chaperonin TRiC/CCT

**Fig. 5:**
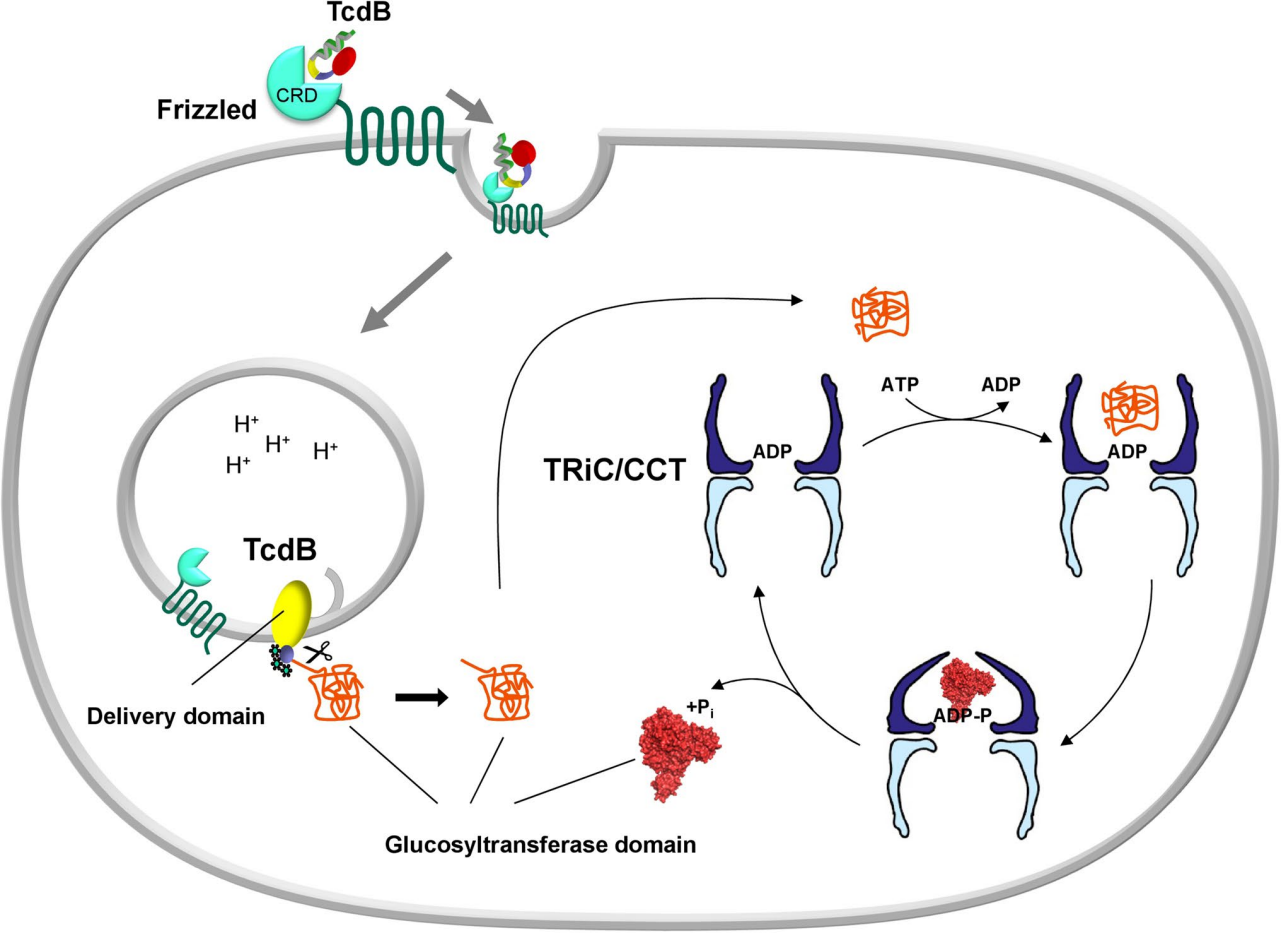
Model of the action of the chaperonin TriC/CCT in TcdB up-take and action. TcdB binds to its receptor Frizzled and is endocytosed. At low pH of endosomes at least GTD and CPD od TcdB are translocated into the cytosol. Most likely, translocation occurs as a single chain. In the cytosol, GTD is refolded with the help of the chaperonin TriC/CCT. The chaperonin has a double cage-like structure and itsaction depends on hydrolysis of ATP. Schema modified from (Russmann et al. 2012)

**Fig. 6:**
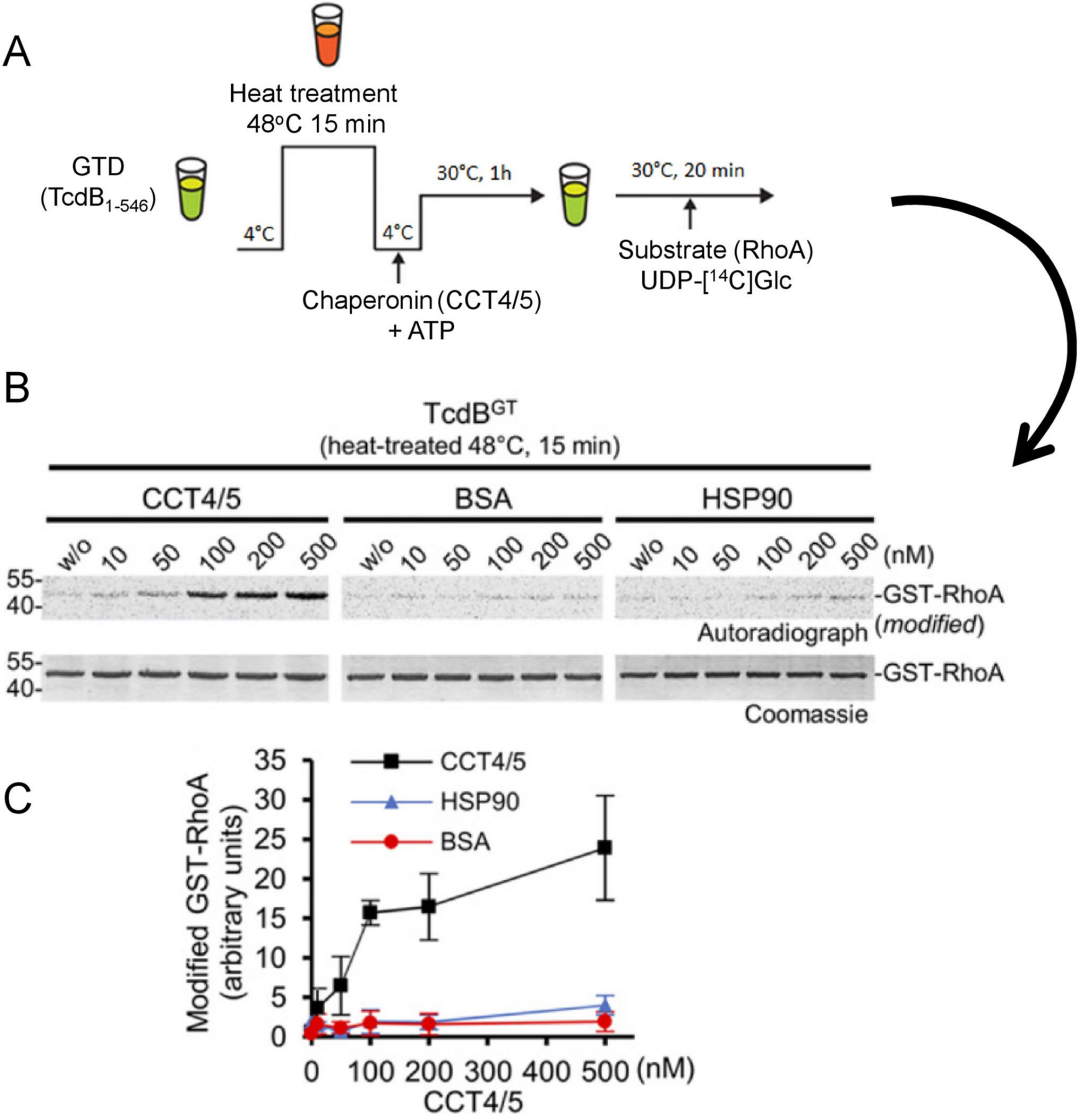
Involvement of chaperonin TriC/CCT in stabilization and refolding of the glucosyltransferase domain of TcdB. **A**. The experimental procedure to show the role of chaperonin is given. The glucosyltransferase domain GTD (residues 1–546) was heated for 15 min at 48 °C, then the chaperonin (CCT4/5) was added together with ATP and the mixture remained for 1 h at 30 °C. Then, the glucosyltransferase activity was studied by addition of the substrate RhoA and radioactively labeled UDP-glucose as a sugar donor. **B**. The autoradiograph shows that CCT4/5 stabilized (refolded) GTD in a concentration-dependent manner. BSA or the HSP90 chaperone were without effects. **C**. Quantification and statistics of the experiment given under B. (Data from Steinemann et al. 2018)

Recently, it was shown that chaperonin TRiC/CCT (TCP-1 ring complex (TRiC)/chaperonin containing TCP-1) is involved in the up-take and refolding of TcdB and other glucosylating toxins (Steinemann et al. 2018). TRiC/CCT is a molecular machine, which is involved in folding of numerous newly synthesized eukaryotic proteins (Yam et al. 2008; Lopez et al. 2015). The chaperonin is essential for the folding of many cytoskeleton proteins, including actin and tubulin (Chen et al. 1994; Leroux and Hartl 2000). Nearly 10% of cytosolic proteins seem to interact with TRiC/CCT (Balchin et al. 2016). TRiC/CCT consists of 2 ring-shaped oligomeric champers with 8 non-identical subunits in each ring. The 2 ring-complex binds unfolded proteins and promotes their folding in the chambers in an ATP-dependent manner (Russmann et al. 2012) (Fig. 5). Figure 6 gives a simple experiment showing that the presence of a mixture of CCT4 and 5 subunits protects the glucosyltransferase domain of TcdB from heat inactivation. Notably, the chaperon HSP90, which is essential for translocation and reactivation of ADP-ribosylating toxins, exhibited no effects (Haug et al. 2003). This protecting (refolding) effect of CCT on GTD is ATP-dependent. Two findings indicate that TRiC/CCT plays a crucial role in TcdB-induced intoxication. First, the chaperonin inhibitor HSF1A (Neef et al. 2014) blocks the intoxication of cells by TcdB. By contrast the effects of ADP-ribosylating toxins like *C. botulinum* C2 toxin are not inhibited. Secondly, knock-down of CCT5 by siRNA protects cells against TcdB toxicity but not against the ADP-ribosylating C2 toxin. Importantly, the chaperonin system seems to interact with all types of glycosylating toxins not only with clostridial toxins like TcdA and TcdB, but also with, e.g., PaTox, which is a tyrosine-modifying GlcNAc-transferase (Jank et al. 2013; Steinemann et al. 2018). Various models of toxin translocation from toxin types other than glucoslating toxins suggest that the delivered toxins occur in the cytosol as a linear chain, comparable as they are synthesized at ribosomes. At this stage, the chaperones may act to facilitate toxin folding. Moreover, it is likely that the support of refolding of toxin domains and subdomains in the cytosol affects the dynamics and directionality of toxin translocation in a manner characterized as entropic pulling (Goloubinoff and De Los Rios 2007).

## Modification of Rho proteins

The discovery in 1994 that *C. difficile* toxins suppress subsequent ADP-ribosylation of Rho proteins in the cell lysate let us to the hypothesis that TcdB acts on Rho proteins (Just et al. 1994). Subsequently, TcdA and TcdB have been characterized as mono-*O*-glucosyltransferases that transfer a glucose onto RhoA using UDP-glucose as a sugar donor (Just et al. 1995a, 1995b). The best characterized protein substrates of TcdB are RhoA/B/C, Rac1 and Cdc42 which are glucosylated at Thr-37 in RhoA/B/C and the homologous Thr-35 in Rac/Cdc42. The related α-toxin from *C. novyi* (TcnA) and lethal toxin from *C. sordellii* (now called *Paeniclostridium sordellii*) (TcsL) differ from TcdA and TcdB, as TcnA GlcNAcylates Rho proteins (Selzer et al. 1996) and lethal toxin preferably glucosylates Ras proteins (Just et al. 1996). Using mass spectrometry methods, an arrow of additional Rho/Ras GTPases have been identified to be glucosylated by the LCTs (Genth et al. 2018; Zeiser et al. 2013). In all cases, Rho GTPases are inactivated by toxin-caused glucosylation.

**Fig. 7:**
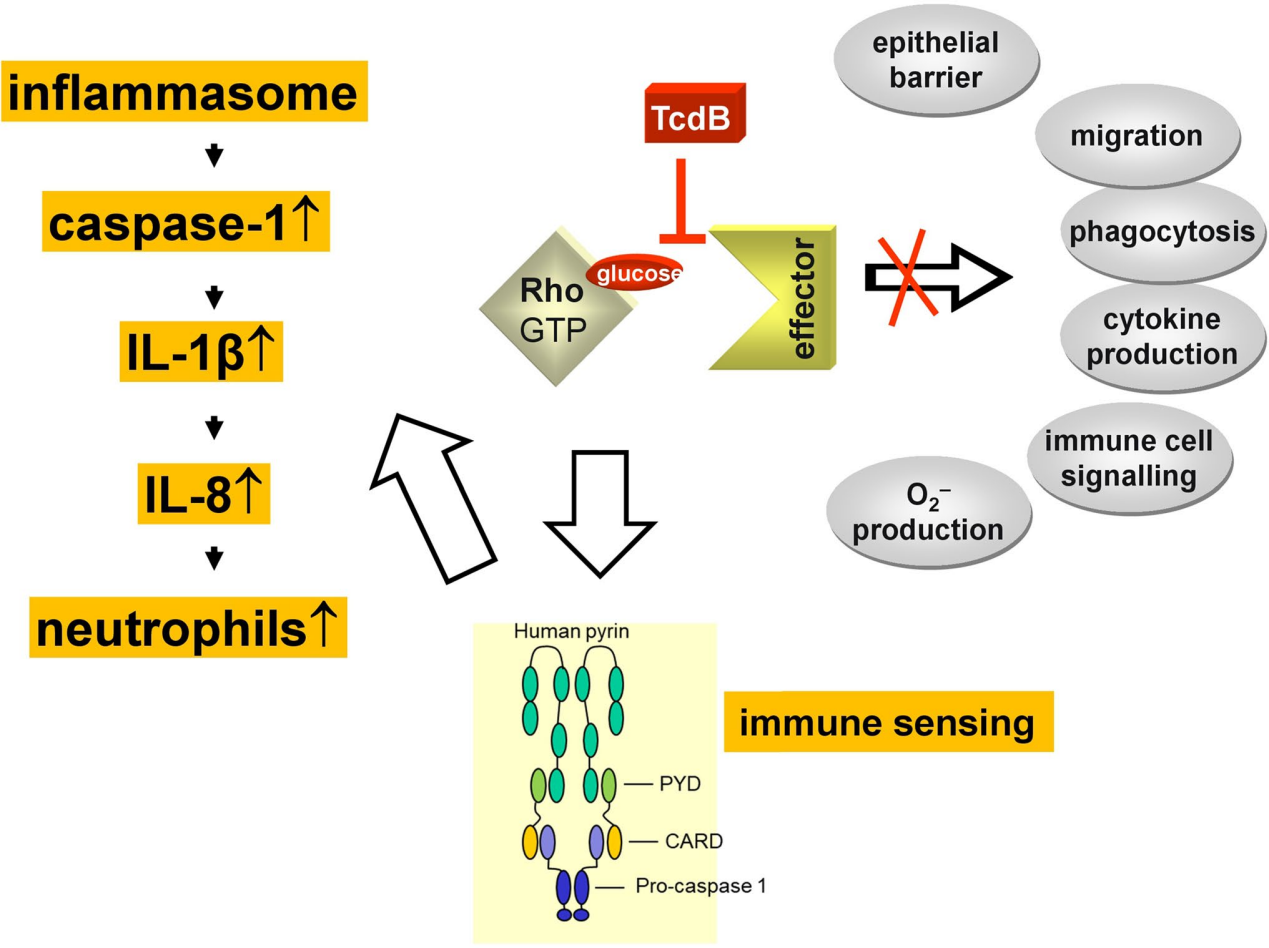
Functional consequences of Rho inactivation by TcdB-induced glucosylation. Glucosylation of Rho inhibits the interaction of this switch protein with numerous effectors, thereby epithelial barrier functions, cell migration, phagocytosis, cytokine production, immune cell signaling and O^2−^ production is blocked. On the other hand, Rho inhibition causes pyrine inflammasome activation, eventually resulting in release of IL-1β with subsequent IL-8 release and attraction of neutrophil leukocytes

Rho proteins are GTP-binding proteins and are regulated by GTPase cycle (Cherfils and Zeghouf 2013). They are inactive in their GDP-bound form and are activated after release of GDP (rate-limiting step) and binding of GTP. GTP-binding causes changes in the so-called switch regions of the Rho proteins thereby allowing activating interactions with numerous effectors. Three groups of regulatory proteins control the activity state of Rho proteins: guanine nucleotide exchange factors (GEFs) activate Rho proteins, GTPase-activating proteins (GAPs) inactivate Rho proteins and guanine nucleotide dissociation inhibitors, which mainly keep Rho in an inactive state in the cytosol (Lamarche and Hall 1994; Schmidt and Hall 2002; Cherfils and Zeghouf 2013). Because Rho proteins are involved in numerous regulatory pathways (Burridge and Wennerberg 2004), multiple effects are the consequences of the toxin-induced glucosylation of Rho in Thr37 (Rac and Cdc42 in Thr35) (Aktories 2011) (Fig. 7). Toxin-induced inactivation of Rho proteins induces changes in cell morphology, redistribution of the actin cytoskeleton and loss of stress fibers (Ottlinger and Lin 1988). Many effects depend on the cell type and their different natural equipment of Rho subtype proteins. Thus, besides major effects on the cytoskeleton, on cell attachment and cell contacts, TcdB blocks cell proliferation and induces different types of cell death including necrosis, apoptosis, or pyroptosis (Aktories et al. 2017a). Basic functions of immune cells like migration, adhesion, secretion, and superoxide production are inhibited or strongly affected. Interestingly, inactivation of Rho proteins also causes activation of some immune cells resulting in activation of the pyrin inflammasome with pyroptosis and release of IL-1β (Xu et al. 2014).

## *C. difficile* ADP-ribosyltransferase CDT

**Fig. 8:**
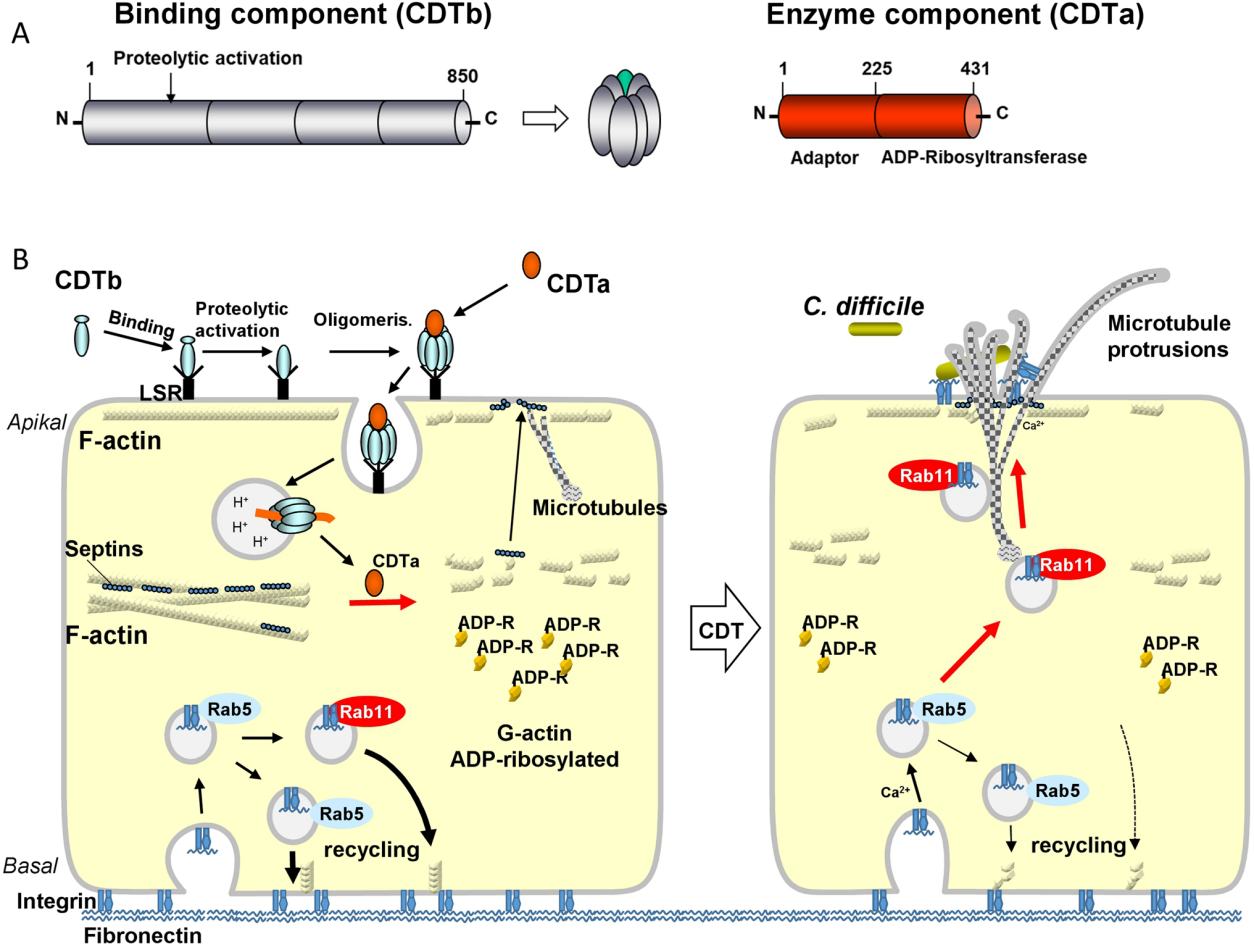
Structure and actions of *C. difficile* ADP-ribosyltransferase CDT. A. CDT is a binary toxin and consists of two separated toxin components, the binding component CDTb and the enzyme component CDTa. CDTb is proteolytically activated and forms heptamers. CDTa has an adaptor domain at the N-terminus and an enzyme domain (ADP-ribosyltransferase) at the C-terminus. B. Model of the actions of CDT. Left cell: CDTb binds to LSR, is proteolytically activated and forms hepatmers. So far it is unclear, whether the activation step is before or after receptor binding. The heptamers bind the enzyme component CDTa. The receptor-toxin complex is endocytosed. At low pH of endosomes CDTb forms pores and translocates CDTa into the cytosol. Here, CDTa ADP-ribosylated G-actin and inhibits the polymerisation of actin. Depolymerisation of submembranous F-actin allows formation of microtubule-based protrusions and releases septins from F-actin, which guide the microtubules into the protrusions. The lower part shows the recycling of vesicles with integrin and bound fibronectin. Right cell: CDTa-induced ADP-ribosylation of actin results in misguiding of vesicles (Rab11-associated vesicles) to the apical membrane, where fibronectin is released. Microtubule-based protrusions and fibronectin enhance binding of *C. difficile* bacteria

As mentioned above, many hypervirulent strains of *C. difficile* produce the ADP-ribosylating toxin CDT (Popoff et al. 1988). CDT belongs to the family of binary toxins, which are characterized by two separated toxin subunits (Barth et al. 2004). One subunit is an ADP-ribosyltransferase and represents the biologically active part (Fig. 8). The second subunit is involved in receptor-binding and translocation of the enzyme domain into the cytosol of target cells. CDT exhibits structural and functional similarity with *C. botulinum* C2 toxin, *C. perfringens* iota toxin, and *C. spiroforme* toxin (Aktories et al. 2011, 2012, 2017b). In fact, its discovery was mainly stimulated by research on the binary actin-modifying *C. botulinum* C2 toxin (Aktories et al. 2018). Important to mention that the binding/translocation domain of all these actin-modifying toxins are very similar to the binding component of anthrax toxin (Young and Collier 2007). All these binding components of the binary toxins are activated as monomers by proteolytic cleavage and, subsequently, heptamerize, resulting in pore-formation at low pH of endosomes. The receptor of CDT is lipolysis-stimulated lipoprotein receptor (LSR) (Papatheodorou et al. 2011). In addition, *C. perfringens* and *C. spiroforme* toxins bind to LSR but not *C. botulinum* C2 toxin or anthrax toxin. LSR appears to be involved in lipoprotein clearance but has also an important role in tricellular tight junctions (Furuse et al. 2012).

## The actions of CDT

**Fig. 9:**
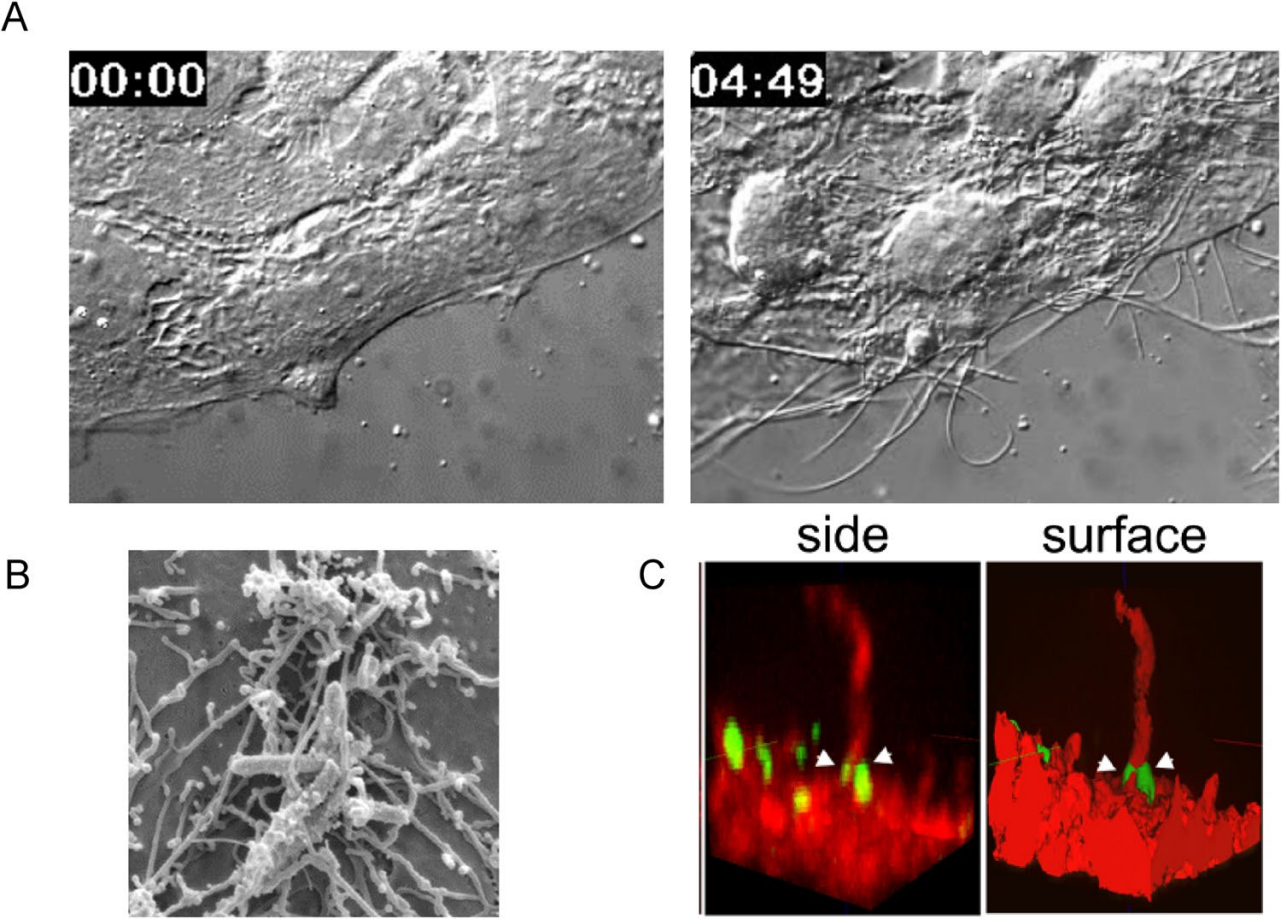
Effects of *C. difficile* ADP-ribosyltransferase CDT on Caco-2 cells. A. Addition of CDT to Caco-2 cell culture results in formation of long microtubule-based cell protrusion (left, control; right CDT). B. The net, formed by microtubule-based cell protrusions, increases the adherence of *C. difficile* bacteria C. Septins (yellow and arrow head) are involved in guiding of microtubles at the membrane. Septins form a funnel-like structure for microtubles. Data from Schwan et al. 2009 and from Nölke et al., 2016

CDT ADP-ribosylates actin at arginine-177. The same acceptor amino acid is also shared by the related *C. botulinum* C2-toxin (Aktories et al. 1986). The functional consequences of ADP-ribosylation of actin at arginine-177 have been studied in detail in the late eighties of the last century. ADP-ribosylated actin does not polymerize. In addition, ADP-ribosylated actin acts as a plus-end actin-capping protein to inhibited polymerization of non-modified actin (Aktories et al. 2011, 2018). Moreover, previous studies, mainly directed by Carsten Schwan in my laboratory, revealed that depolymerization of F-actin results in formation of microtubule-based cell protrusions (Schwan et al. 2009) (Figs. 8, 9). Here, another constituent of the cytoskeleton (so-called fourth component of the cytoskeleton) came into play: Septins, which are GTP-binding proteins and able to polymerize, are involved in microtubule protrusion formation and guidance of protrusions (Nolke et al. 2016). The CDT-induced microtubule-based protrusions appear to enhance the adhesion of clostridia to epithelial gut cells. Furthermore, the protrusions allow retrograde and antegrade vesicle trafficking. CDT-induced redistribution of the actin cytoskeleton and formation of microtubule-based protrusions alter recycling of Rab-5 and Rab-11-associated vesicles at the basolateral membranes of epithelial cells. Subsequently, vesicles are misguided to the apical membrane into the CDT-induced protrusions, where fibronectin is released by the vesicles (Schwan et al. 2014) (Fig. 8). Release of fibronectin and formation of the tentacles by CDT enhances clostridia attachment. The functions of CDT in infection are still not well understood. Fifteen to twenty-five percent of *C. difficile* strains (very often hypervirulent strains) produce in addition to TcdA and TcdB the binary CDT. Very few *C. difficile* strains exist, which produce CDT only. In animal models, CDT alone may preferentially affect the small intestine and less the large intestine (Geric et al. 2006). Effects of CDT on immune cells have been reported (Nibbering et al. 2021) but the precise effects of CDT on immune cells are not clear. In this respect, one should also consider experiments with *C. botulinum* C2 toxin on various types of blood cells. C2 toxin shares the identical intracellular action with CDT. Here, it was shown that actin disruption by C2 toxin facilitates exocytosis and O^2−^ formation (Wenzel-Seifert et al. 1997) but inhibited neutrophil migration (Norgauer et al. 1988). A severalfold increase in diacylglycerol formation and sustained elevation of cytosolic calcium occurred after C2 treatment of human neutrophils (Grimminger et al. 1991). Because the CDT-receptor LSR is also expressed in leukocytes (Expression Atlas EMBL-EBI), similar effects are likely for CDT.

## Treatment of CDI

**Fig. 10:**
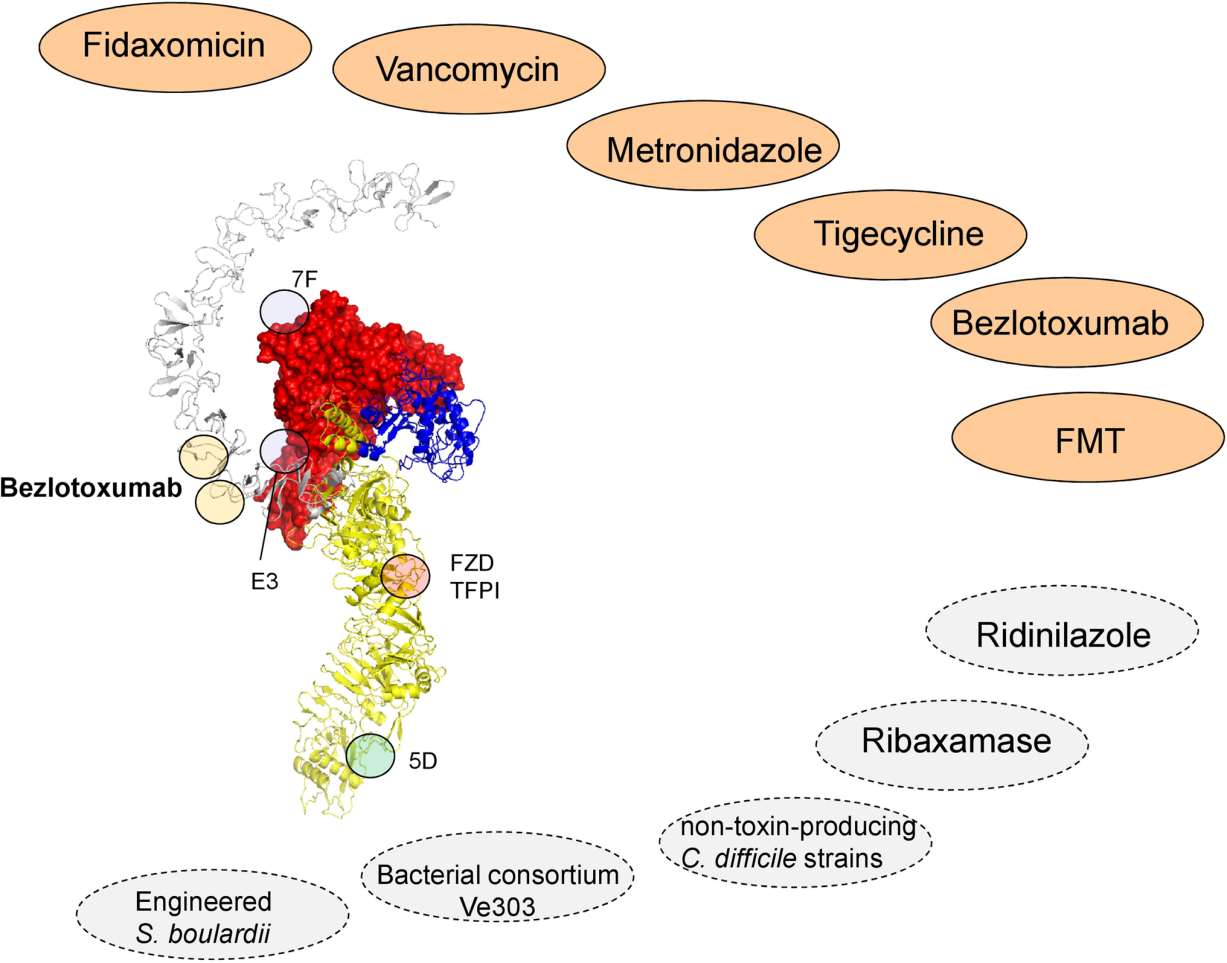
Treatment options for diseases caused by *C. difficile* infection. Colored ovates represent approved treatments including the antibiotics fidaxomicin, vancomycin, metronidazole, and tigecycline, the anti-TcdB-antibody bezlotoxumab and fecal microbiota transplantation (FMT). The dotted ovates show proposed future treatment options (for details see text). The structure of TcdB shows the target sites of Bezlotoxumab, the binding sites of three neutralizing monovalent antibody E3, 7F, 5D and the binding region of FZD and TFPI

Treatment of CDI is an ongoing challenge (Fig. 10). This is especially true in view of the high rate of recurrent CDIs, which occurs in about 25% of cases of successful treatment of an initial colitis phase (McFarland et al. 1999). In about 20% of patients, the stop of the administration of the CDI-inducing antibiotics results in termination of diarrhea within 2–3 days. Up to recently oral vancomycin and metronidazole were standard therapy (for example (Lubbert et al. 2014)). This is changing. For the initial phase of non-severe CDI, fidaxomicin (200 mg, twice daily) is now recommended by ISDA (Infection Diseases Society of America) and ESCMID (European Society of Clinical Microbiology and Infectious Diseases) for 10 days (Johnson et al. 2021, van Prehn et al. 2021). Fidaxomicin is especially recommended, when risk factors for recurrent disease are present (e.g., age, prior CDI episode, PPI therapy). Alternatively (if fidaxomicin is not available), oral vancomycin (125 mg, four times daily, 10 days) is indicated. Metronidazole (500 mg, three times daily) is only recommended, if the above drugs cannot be used. Some properties of fidaxomicin are of interest. It is a macrolide antibiotic, which inhibits the bacterial RNA polymerase. Moreover, it exhibits some specificity for *C. difficile*. Recently, the reason for this specificity was unraveled. A single amino acid residue in the *C. difficile* RNA polymerase sensitizes fidaxomicin’s narrow-spectrum activity. This amino acid is absent in most gut microbiota (Cao et al. 2022).

Various antibodies have been selected for anti-toxin treatment of CDI. While the anti-TcdA antibody Actoxumab turned out to be ineffective, Bezlotoxumab, a monoclonal TcdB antibody directed against the N-terminal part of the CROPS domain, is more effective and approved for treatment in many countries. It is recommended for recurrent CDI. However, the evidence of benefit of adding the antibody is still not clear. The advantage of the addition of bezlotoxumab in recurrent CDI as compared to standard treatment (Modify trial) resulted in a reduction of the recurrence rate from 28 to 17% (Wilcox et al. 2017).

Treatment for severe CDI (characterized (ESCMID) by fever (> 38.5°), marked leukocytosis (> 15 × 10^9^ /L), and rise (> 50%) in serum creatinine) is similar, as given above, with vancomycin or fidaxomicin (van Prehn et al. 2021). Metronidazole i.v. and tigecycline are additional options, although with very limited evidence from randomized control trials (RCTs). In severe-complicated or fulminant CDI (characterized by septic course and/or ileus, toxic megacolon or bowel perforation), early consultation of a surgeon is good clinical practice. In addition, fecal microbiota transplantation (FMT) may be of great value (Song et al. 2022).

### Fecal microbiota transplantation (FMT) and the problem of recurrent CDI

For multiple recurrent CDI, FMT is an efficient option. The first reported treatment with FMT of pseudomembranous colitis was already in 1953 (Eiseman et al. 1958). Now, FMT has been proven to be highly efficient in recurrent CDI. In a controlled clinical study, resolution of symptoms were observed in 31%, whereas FMT (via a nasoduodenal tube) resulted in resolution in 80% of cases (van Nood et al. 2013). However, this was a very small study. A recent meta-analysis suggested less effectiveness of this treatment (66.4–85.7% resolution) (Tariq et al. 2019). The underlying therapeutic mechanism of FMT is still not clear and might involve 1. a direct killing of *C. difficile*, 2. nutrient competition between FMT species and *C. difficile*, and 3. production of crucial gut metabolites, which inhibit *C. difficile* development from spores, growth, and toxin production or may promote increased intestinal barrier functions. However, the risk of FMT as a live biotherapeutic has to be considered. This is especially problematic in immunocompromised patients (Severyn et al. 2019).

In 2019, two FMT recipients (one died) developed severe illness caused by transplantation of multidrug resistant *E. coli* (DeFilipp et al. 2019). In 2020, FDA recalled a FMT preparation, because six patients were infected with Shiga toxin-producing *E. coli* (STEC) from the donors’ stools; two patients died from diarrhea associated with STEC infections (Buckley et al. 2022).

## Future treatment developments

What are further developments? Another narrow spectrum agent, ridinilazole, is presently studied in clinical trials (Collins and Riley 2022). The molecular mechanism of this compound is different, as it appears to inhibit septum formation of clostridia and to impair cell division of *C. difficile* (Basseres et al. 2016). Another interesting approach to prevent CDI in patients, who are i.v.-treated with the cephalosporine Ceftriaxone, is the oral administration of the non-absorbed β-lactamase ribaxamase (Kokai-Kun et al. 2019). Intravenous ceftriaxone is biliary excreted and destroys the gut microbiome. This may be prevented by the β-lactamase. In addition, in the antibody field several different approaches were tried. For example, *Saccharomyces boulardii* was engineered to constitutively secrete a neutralizing, tetraspecific antibody composed of single-domain variable fragments of heavy-chain antibodies against both TcdA and TcdB. This preparation was able to protect against primary and recurrent CDI in both prophylactic and therapeutic mouse models of disease but not in hamsters (Chen et al. 2020).

Whether probiotics are helpful for prophylaxes or therapy of CDI is an ongoing question. ESCMID does not recommend routine administration of probiotics to prevent CDI. Of great interest was the study by Suez et al. (Suez et al. 2018), showing that probiotics may impair the reconstitution of the gut microbiome after antibiotic treatment, while autologous FMT enhanced the reconstitution. An exciting approach is the recent design and analysis of a well-defined microbial community of eight commensal strains of Clostridia (named VE303), which were isolated from healthy donors. This product with the eight strains of VE303 were able to inhibited *C. difficile* growth in vitro. Moreover, VE303 strains colonized the gut of healthy volunteers after vancomycin pretreatment and promoted production of secondary bile acids and of short fatty acids, which both block spore germination (Dsouza et al. 2022).

Finally, another approach is the administration of non-toxin-producing *C. difficile* strains against primary and recurrent episodes of CDI (Shim et al. 1998; Gerding et al. 2015). This is apparently effective, however, it has been suggested that the entire pathogenicity locus of *C. difficile* might be transferred from a toxigenic to a non-toxigenic strain (Brouwer et al. 2013), which would not be advantageous.

Taken together, the biology of *C. difficile* and the treatment of CDI is an exciting field especially from the pharmacological point of view. I believe and it is shown here that the development of biochemical and molecular pharmacology exhibited major impact on research and development in the discipline of toxinology. Moreover, my view is that the field of bacterial toxins is still not fully exploited and conceal treasures for the use of toxins as pharmacological tools and drugs.

## Data Availability

Not applicable.

## Footnotes

1 Please note, only a very limited number of references are cited. Many important publications from other laboratories are not cited, which is due to the narrative character of this review.
